# Effect of *Bombyx mori* on the Liver Protection of Non-Alcoholic Fatty Liver Disease Based on In Vitro and In Vivo Models

**DOI:** 10.3390/cimb43010003

**Published:** 2021-04-28

**Authors:** Miey Park, Chaewon Kang, Hae-Jeung Lee

**Affiliations:** 1 Department of Food and Nutrition, College of BioNano Technology, Gachon University, Gyeonggi-do 13120, Korea; mieyp@naver.com (M.P.); kcwon9512@hanmail.net (C.K.); 2 Institute for Aging and Clinical Nutrition Research, Gachon University, Gyeonggi-do 13120, Korea

**Keywords:** *Bombyx mori* (silkworm), non-alcoholic fatty liver disease (NAFLD), AMP-activated protein kinase (AMPK), acetyl-CoA carboxylase (ACC)

## Abstract

Edible insects, *Bombyx mori* (silkworm; SW), which feed on mulberry leaves, have been consumed by humans for a long time as supplements or traditional medication. Non-alcoholic fatty liver disease (NAFLD) is a liver metabolic disorder that affects many people worldwide. We examined the hepatoprotective effects of SW using in vitro and high-fat and high-fructose (HFHF) diet-induced obese in vivo model mice by real-time PCR, immunoblot analysis, and fecal microbiota analysis. SW significantly reduced lipid accumulation and expression of the lipogenic genes in HepG2 cells and the livers of HFHF-induced mice. SW caused significant reductions in triglycerides, and total cholesterol in serum and upregulation of fatty acid oxidation markers compared to the HFHF group. Besides, SW significantly induced phosphorylation of AMPK and ACC in both models, suggesting roles in AMPK activation and the ACC signaling pathway. Furthermore, the gut microbiota analysis demonstrated that SW treatment reduced Firmicutes to Bacteroidetes ratios and the relative abundance of the Lachnospiraceae family compared to HFHF-induced obese mice. These results provide a novel therapeutic agent of hepatoprotective effects of SW for non-alcoholic hepatic steatosis that targets hepatic AMPK and ACC-mediated lipid metabolism.

## 1. Introduction

Non-alcoholic fatty liver disease (NAFLD) is a metabolic disorder of the liver that affects a large number of people worldwide [1]. As the prevalence of obesity, diabetes, and metabolic syndrome among the general public increases, NAFLD is increasing proportionately [2]. NAFLD is a chronic liver disease involving the accumulation of triglyceride (TG) in liver cells, with fat mass causing steatosis through fatty peroxidation reactions [3,4]. Excess lipid accumulation is the result of abnormal fatty acid metabolism and leads to elevated hepatic oxidative stress [5]. Furthermore, NAFLD encompasses a broad spectrum of hepatic injuries ranging from liver fibrosis to hepatocellular carcinoma [6]. Currently, treatment is limited to the suggestion of lifestyle changes, as no pharmacologic treatments have been approved for NAFLD [7].

Edible insects are nutritious foods rich in protein, amino acids, various vitamins, and trace elements [8]. As the global population increases, palatable insects may be further developed as food ingredients [9]. Among them, *Bombyx mori* (silkworm; SW) is considered a promising candidate among edible insects, as it has been consumed by humans for a long time [10]. Steam-cooked SW powder is used as a traditional Korean medicine and has been studied for reducing blood glucose and obesity by the National Sericulture and Entomology Research Institute (NSERI) and National Institute of Crop Science (NICS) in Korea. *Bombyx mori* is a monophagous and nocturnal moth belonging to the Bombicidae family. Commonly known as silkworm, it has been used for several decades in China and domesticated worldwide as a popular medicine. As listed in the well-known old Chinese pharmacological script “Honzo-komokii” adult moths can be used as an aphrodisiac [11,12]. Besides, larvae and feces of this insect were used as an antifever in certain areas [13]. In Chinese medicine, the use of male moths has also been reported to treat sterility [14]. The freeze-dried powder of silkworm larvae has been used as an additive to protect the liver and avoid ailments among older people [15]. Additionally, processed silkworm larvae are used in special diets for diabetics and heart patients [16]. Tar obtained from silkworm pupae with proven and superior antibacterial and antihistaminic activities has also been documented compared to plant sources [17]. Oil extracted from pupae has been used to treat hepatic ailments and blood complications [18]. Silkworm pupae protein is valued higher than fish, soybean, and beef protein [19]. According to several recent studies, SW is considered an economically viable material for conventional medication [20]. Rye et al. reported that SW had beneficial effects on diabetics [21], and improved control of blood glucose levels was verified in subsequent research [22]. Recently, products made from mulberry leaf and SW have become popular as supplements for individuals with diabetes mellitus. However, little has been reported on the hepatoprotective effects of SW against NAFLD.

Numerous transcription factors are associated with hepatic lipid metabolism. Sterol regulatory element-binding protein 1c (SREBP-1c) induces hepatic lipogenesis and lipid accumulation [23]. Fatty acid synthesis is catalyzed by acetyl-CoA carboxylase (ACC) and fatty acid synthase (FAS), an enzyme that plays a central role in providing substrates for energy metabolism and is regulated by various nuclear receptors [24,25]. Additionally, peroxisome proliferator-activated receptors (PPARs) are lipid-activated transcription factors that serve as fatty acid receptors and regulators of lipid metabolism and glucose homeostasis [26]. PPAR alpha (PPARα) activation regulates hepatic fatty acid and lipoprotein metabolism [27], while PPAR gamma (PPARγ) is necessary for adipogenesis [28]. Besides, AMP-activated protein kinase (AMPK) is a regulator of cellular energy homeostasis, inhibits glucose production by the liver, increases fatty acid oxidation, and decreases lipogenic gene expression phosphorylation of the master transcriptional regulator of lipogenesis [29,30].

Based on these results, we investigated the hepatoprotective effects of SW in FFA-treated HepG2 cells and high fat and high fructose (HFHF)-induced obese mice, and report therapeutic effects of SW on the improvement of NAFLD. To our knowledge, this is the first study into the ameliorative effect of NAFLD of SW.

## 2. Materials and Methods

### 2.1. SW Powder Preparation for Metabolite Analysis

Steam SW powder was purchased from the Shin Young-deok sericulture association (Yeongdeok-gun, Gyeongbuk, Korea). The extraction method for SW metabolites described by Li et al. [31] was used with some modifications. Each SW powder (100 mg) was extracted with 80% methanol (1 mL) and 10 μL of internal standard solution (2-chloro-phenylalanine, 1 mg/mL in water) using an MM400 mixer mill (Retsch^®®^, Haan, Germany) at a frequency of 30 s-1 (10 min), followed by sonication (10 min). Subsequently, the extracted samples were centrifuged at 15,000 rpm (10 min) at 4 °C and the supernatants were filtered through 0.2-μm polytetrafluoroethylene (PTFE) filters (Chromdisc, Daegu, Korea). The filtered supernatants were completely evaporated using a speed vacuum concentrator (Biotron, Seoul, Korea).

### 2.2. Gas Chromatography-Time of Flight-Mass Spectrometry Analysis (GC-TOF-MS)

GC-TOF-MS was conducted as described in our previous study [32]. For analysis, all dried samples were oximated with 50 μL of methoxyamine hydrochloride (20 mg/mL in pyridine, 90 min at 30 °C) and silylated with 50 μL of N-methyl-N-(trimethylsilyl) trifluoroacetamide (MSTFA, 30 min at 37 °C). The derivatized sample (1 μL) was injected into the GC-TOF-MS instrument at a split ratio of 10:1.

### 2.3. Ultrahigh Performance Liquid Chromatography-Linear Trap Quadrupole-Orbitrap-Mass Spectrometry (UHPLC-LTQ-Orbitrap-MS/MS) Analysis

UHPLC-LTQ-Orbitrap-MS/MS analysis was conducted as described previously [32]. Prior to UHPLC-LTQ-Orbitrap-MS/MS analysis, all dried samples were redissolved in 80% methanol.

### 2.4. Cell Culture and Free Fatty Acid (FFA) Treatment

Human HepG2 hepatocytes were attained from the ATCC (Manassas, VA, USA). HepG2 cells were cultured in DMEM (10% fetal bovine serum) with 1% antibiotics (Thermo Fisher Scientific, Waltham, MA, USA) and incubated at 37 °C and 5% CO_2_ in a humid chamber. A 1-mM mixture of FFAs (oleic acid/palmitic acid, 2:1) was dissolved in 2-propanol. Cells at 80% confluence were starved with serum-free medium supplemented with 1% bovine serum albumin (BSA). Only 1% BSA was applied to control cells.

### 2.5. Cell Cytotoxicity Detection and Oil Red O Staining

Cell cytotoxicity was performed using the Cell Counting Kit-8 (CCK-8) assay (Dojindo Molecular Technologies, Rockville, MD, USA). HepG2 cells were seeded into plates (5 × 104 cells/well) and incubated 24 h at 37 °C in a CO_2_ incubator. After incubation, the medium was replaced with serum-free DMEM containing 1 mM FFA and 1% BSA following treatment with various concentrations of SW (0, 25, 50, 100, and 200 µg/mL) at 37 °C for 24 h. Then, CCK-8 assay solution (10 µL/well) was added and incubated for a further 2 h at 37 °C in the dark. The experiment was performed in triplicate. The absorbance (450 nm) was measured using a microplate spectrophotometry system (Biotek Inc., Winooski, VT, USA). Cells were fixed in 10% formalin and stained with Oil Red O working solution (30 min) at room temperature. After washing three times with distilled water, cells were observed under a microscope and images were captured. After observation of intracellular lipid accumulation, 1 mL of 100% isopropanol was added and absorbance was measured at 500 nm. Values are expressed as a percentage relative to the control.

### 2.6. Experimental Animals and Diets

Five-week-old C57BL/6J male mice were obtained from Central Laboratory Animal Co. (Seoul, Korea) and housed under controlled temperature (20–25 °C) and humidity (50–55%) conditions with a 12 h/12 h light–dark cycle. After acclimatization, the mice were divided into randomly: a control group given a normal diet (AIN-93G, Research Diets, Inc., New Brunswick, NJ, USA) and an NAFLD-induced group given a 45% high-fat diet (D12451, Research Diets, Inc., New Brunswick, NJ, USA) with high-fructose (10%) drinking water for 8 weeks. After 8 weeks, the mice were divided into five groups randomly (*n* = 7 per group): a normal diet control (NC) group, which received distilled phosphate-buffered saline (PBS) only, a high-fat and high-fructose diet (HFHF) group administered distilled PBS, an HFHF group treated with 1% silymarin (silymarin has been widely studied for hepatoprotection areas) in PBS (positive control, PC), an HFHF group given 100 mg/kg SW powder in PBS (S100), and an HFHF group administered 200 mg/kg SW powder in PBS (S200). SW treatment was supplemented with oral administration of the same volumeline (200 μL) for 8 weeks. Food consumption was monitored every two days, and body weight was measured weekly. All animal experiments were handled according to protocols approved by Gachon University for the care and use of laboratory animals (ref. no. GIACUC-R2019004, 28 January 2020).

### 2.7. Blood and Tissue Sample Preparation and Histological Analysis

After the experiment, the mice were sacrificed through CO_2_ asphyxiation. Blood was collected and centrifuged at 3000 rpm for 15 min at 4 °C to obtain the serum, which was stored at −80 °C. After collection of blood, liver and adipose tissues were removed and stored immediately at −80 °C for subsequent use. Liver and fat tissues were fixed in 10% formalin. For histological observation, tissue sections (3–4 μm) were stained with hematoxylin and eosin (H&E). An Olympus Provis AX 70 microscope (Olympus, Tokyo, Japan) was used, and images were acquired with a Nikon camera (Nikon, Tokyo, Japan) and quantified using ImageJ software (National Institutes of Health, Bethesda, MD, USA).

### 2.8. Biochemical Analysis of Liver Tissues

The serum aspartate aminotransferase (AST) and alanine aminotransferase (ALT) were measured using ALT and AST assay kits (Asanpharm, Hwaseong, Korea). Lipid peroxidation (hepatic malondialdehyde, MDA) was calculated using the clear supernatant based on measurement of absorbance at 532 nm and expressed as nmol MDA per weight (g) of liver tissue. The liver tissue (0.1 g) was homogenized with 1 mL of KCl (1.15%) (Sigma-Aldrich, Co., St. Louis, MO, USA). Subsequently, 0.2 mL sodium dodecyl sulfate (SDS, Sigma-Aldrich, Co.), 1.5 mL acetic acid (20%), 1.5 mL aqueous 2-thiobarbituric acid (TBA, 0.8%,), and 0.7 mL distilled water were added sequentially. After incubation at 95 °C for 30 min followed by cooling on ice, 1 mL of distilled water and 5 mL of n-butanol were added to the mixture consecutively, which was vortexed and centrifuged at 4000 rpm for 20 min. Hepatic TG levels were determined according to the method of Folch et al. [33] with modifications, and measured using the TG-S kit (Asanpharm, Hwaseong, Korea). Hepatic total cholesterol (TC) levels were measured using a T-CHO kit (Asanpharm, Hwaseong, Korea).

### 2.9. Quantification of Gene Expression Using Real-Time PCR

Total RNA was extracted from HepG2 cells and mouse hepatocytes using an RNA extraction kit (iNtRON Biotechnology, Gyeonggi-do, Korea). cDNA was synthesized from total RNA (50 ng) using the iScript cDNA synthesis kit (BioRad, Hercules, CA, USA). cDNA was synthesized using real-time PCR with SYBR Green Master Mix (TaKaRa Bio, Otsu, Japan). The primer sets used for amplification are listed in Supplementary Table S3. Expression data were normalized to β-actin as a control.

### 2.10. Immunoblot Analysis

HepG2 cells and mouse hepatocytes were lysed in PRO-PREP™ (iNtRON Biotechnology, Seongnam, Korea). Following quantification, equivalent quantities of each protein sample (40 μg) were loaded onto 10% SDS-polyacrylamide gels and electrophoretically transferred to PVDF membranes. The membranes were blocked (5% skim milk) at room temperature. After washing in Tris-buffered saline with Tween (TBST) three times for 10 min each, the membranes were immunoblotted using the following primary antibodies overnight at 4 °C: CPT-1 (1:1000), PPARα (1:1000), ACC (1:2000), p-ACC (1:10,000), AMPK (1:1000), p-AMPK (1:1000), SREBP-1c (1:1000), FAS (1:1000), PPARγ (1:1000), C/EBPα (1:500), and β-actin. After washing, the blots were incubated with the appropriate secondary antibodies for 1 h at room temperature. After another round of washing in TBST, signals were detected using a Western blot detection kit (Amersham Pharmacia, Little Chalfont, Bucks, UK). The protein bands were visualized with the LAS 500 imager (GE Healthcare Bio-Sciences AB, Uppsala, Sweden).

### 2.11. Fecal Microbiota Analysis

For metagenomics, DNA was extracted using the QIAamp PowerFecal DNA Kit (QIAGEN, Hilden, Germany). The quantity and quality of extracted DNA were measured using the Qubit 4 Fluorometer (Thermo Fisher Scientific, Waltham, MA, USA), NanoDrop One Microvolume UV–Vis Spectrophotometer (Thermo Fisher Scientific, Waltham, MA, USA), and agarose gel electrophoresis. The V3–V4 hypervariable region of bacterial 16S rRNA was amplified with unique 8-bp barcodes, and sequenced on the Illumina MiSeq PE300 platform according to a standard protocol [34].

### 2.12. Data Analysis

All results are expressed as the mean ± standard error (SE) of the mean based on triplicates. Statistical analyses were carried out with GraphPad Prism 5.03 (GraphPad Software, San Diego, CA, USA), using a one-way analysis of variance (ANOVA) followed by Tukey’s post hoc test. Metabolites selected from the GC-TOF-MS and UHPLC-LTQ-Orbitrap-MS results were tentatively identified using various databases through comparison with standard compounds based on retention time, MS fragmentation pattern, and mass spectrum (*m*/*z*). Moreover, we confirmed the MS spectral data for selected metabolites using available databases, including the National Institute of Standards and Technology (NIST) database (Version 2.0, 2011, FairCom, Gaithersburg, MD, USA), Wiley 9, the Human Metabolome Database (HMDB, http://www.hmdb.ca/ (accessed on: 28 August 2020)), in-house libraries, and published studies. Statistical analysis was performed using PASW statistics software (IBM SPSS Inc., Chicago, IL, USA). Comparisons with *p* < 0.05 were considered statistically significant.

## 3. Results

### 3.1. Metabolites Identified through GC-TOF-MS and UHPLC-LTQ-Orbitrap-MS Analyses

To identify the active compounds in SW, we performed GC-TOF-MS and UHPLC-LTQ-Orbitrap-MS analyses. A total of 70 metabolites were identified through GC-TOF-MS and UHPLC-LTQ-Orbitrap-MS in steam-cooked SW powders. The total ion chromatograms from GC-TOF-MS and UHPLC-LTQ-Orbitrap-MS are shown in Figure S1. Thirty metabolites were identified through GC-TOF-MS (Table S1) and 27 metabolites through UHPLC-LTQ-Orbitrap-MS (Table S2) in this study. A total of 10 amino acids, 10 sugars and sugar derivatives, 2 fatty acids, 10 miscellaneous metabolites, and 18 non-identified compounds were analyzed using GC-TOF-MS with a cutoff value of 0.7 and *p*-value < 0.05. In UHPLC-LTQ-Orbitrap-MS analysis, two carboxylic acids, two hydroxybenzoic acids, five phenolic acids, 11 flavonols, six lysophospholipids, and three miscellaneous metabolites were identified (Table S2).

### 3.2. Effects of SW on Cell Viability and FFA-Induced Lipid Accumulation in HepG2 Cells

**Figure 1:**
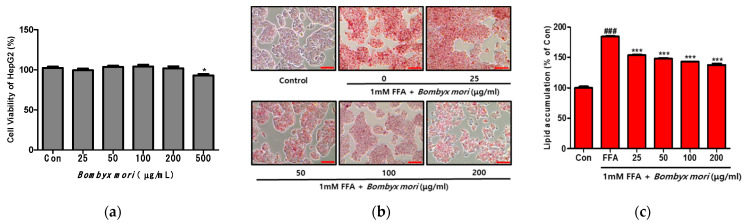
Effects of *Bombyx mori* (SW) on the viability and intracellular lipid accumulation in FFA-induced HepG2 cells. Lipid droplets in HepG2 cells were dyed red (magnification 200×). (**a**) Cell viability of HepG2 cells. * *p* < 0.05 vs. Con. (**b**) Oil Red O staining images of HepG2 cells treated with 1 mM free fatty acids (FFA) and exposed to various concentration of SW with 1 mM FFA for 24 h. (**c**) Quantitative lipid accumulation of Oil Red O contents at 500 nm. Control (Con) cells were incubated with 1% fat-free bovine serum albumin. Data represent means ± SD of three independent experiments. ### *p* < 0.001 vs. Con; *** *p* < 0.001 vs. FFA.

As shown in Figure 1a, SW did not affect the viability of HepG2 cells at concentrations from 25 to 200 μg/mL. In the present study, SW concentrations of 25, 50, 100, and 200 μg/mL were selected for further investigation. HepG2 cells were exposed to FFAs (oleic acid/palmitic acid, 2:1) to induce hepatic steatosis, and intracellular lipid accumulation was visualized after Oil Red O staining (Figure 1b). Then, the HepG2 cells were treated with various concentrations of SW (0, 25, 50, 100, or 200 μg/mL) for 24 h. Control cells (Con) were treated only with 1% BSA. FFA-induced HepG2 cells showed significant increases in lipid droplets and lipid accumulation compared with control cells (Figure 1c). However, lipid accumulation in SW-treated cells decreased in a dose-dependent manner relative to that in FFA-induced cells. These results indicate that SW treatment reduces hepatic lipid accumulation in HepG2 cells.

### 3.3. Effects of SW on Lipogenesis and Fatty Acid Oxidation in FFA-Induced Steatosis

**Figure 2:**
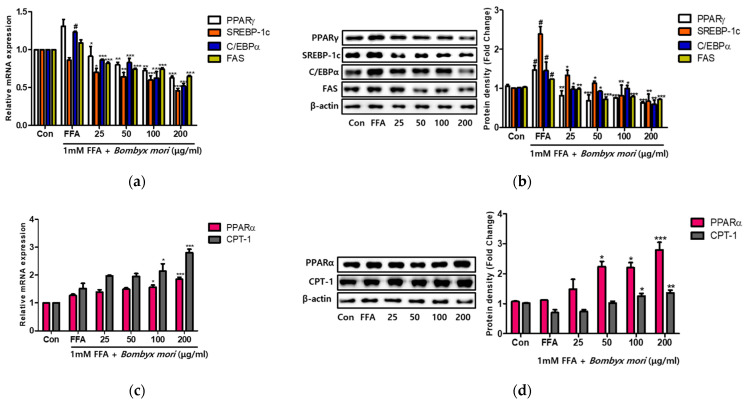
Effects of *Bombyx mori* (SW) on the expression of genes associated with lipogenesis and fatty acid oxidation in FFA-induced HepG2 cells. (**a**) Quantitative real-time PCR analysis of PPARγ, C/EBPα, SREBP-1c, and FAS were normalized by β-actin as an internal control. (**b**) Expression of lipogenesis-related proteins. (**c**) The mRNA expression of PPARα and CPT-1 quantified by real-time PCR and normalized by β-actin as an internal control. (**d**) Immunoblot analysis of PPARα and CPT-1 levels and quantification. PPARγ, peroxisome proliferator-activated receptor gamma; C/EBPα, CCAAT/enhancer-binding protein alpha; SREBP-1c, sterol regulatory element-binding protein 1-c; FAS, fatty acid synthase; PPARα, peroxisome proliferator-activated receptor alpha; CPT-1, carnitine palmitoyltransferase 1. The results of three independent experiments are represented the means ± SE. # *p* < 0.05 vs. Control (Con); * *p* < 0.05, ** *p* < 0.01, and *** *p* < 0.001 vs. FFA.

To examine whether SW could ameliorate FFA-induced steatosis, we evaluated the expression of genes involved in lipogenesis through real-time RT-PCR and immunoblot analyses (Figure 2). The mRNA expression levels of PPARγ, C/EBPα, and FAS were elevated in HepG2 cells treated with 1 mM FFA relative to control cells. However, SW treatment significantly decreased dose-dependently at mRNA expression of PPARγ, C/EBPα, SREBP-1c, and FAS compared to 1 mM FFA only (Figure 2a). Similarly, immunoblot analysis showed that protein levels were also significantly downregulated with SW treatment relative to FFA (Figure 2b). These results suggest that SW attenuates hepatic steatosis through the regulation of lipogenesis in FFA-induced HepG2 cells. Besides, PPARα and CPT-1 genes associated with hepatic fatty acid oxidation were examined. As shown in Figure 2c, SW significantly increased the mRNA expression levels of PPARα and CPT-1 at doses of 100 and 200 μg/mL relative to FFA only induced HepG2 cells. Furthermore, Western blot analysis identified the effects of SW on PPARα and CPT-1 gene expression (Figure 2d).

### 3.4. Effects of SW on the Expression of Phosphorylation of AMPK and ACC in FFA-Induced Steatosis

**Figure 3:**
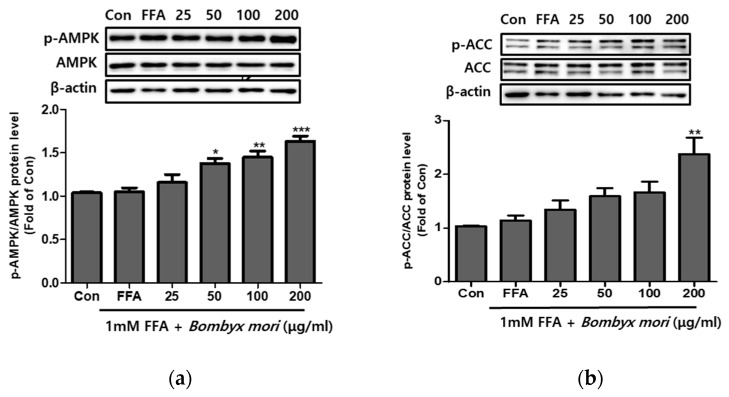
Effects of *Bombyx mori* (SW) on the expression of AMPK and ACC signaling in FFA-treated HepG2 cells. The expression of p-AMPK/AMPK (**a**) and p-ACC/ACC (**b**) were quantified by Western blot analysis. AMPK and ACC were used as a protein loading control of phosphorylated AMPK (p-AMPK) and phosphorylated ACC (p-ACC), respectively. The results from three independent experiments are expressed as the means ± SE. * *p* < 0.05, ** *p* < 0.01, and *** *p* < 0.001 vs. FFA.

Compared with FFA-induced HepG2 cells, SW treatment increased hepatic fatty acid oxidation-related protein levels. Following SW treatment for 24 h, the phosphorylation levels of AMPK and ACC were significantly elevated compared to FFA-only treatment. As shown in Figure 3a,b, SW treatment improved the p-AMPK/AMPK ratio and enhanced p-ACC/ACC in a dose-dependent manner.

### 3.5. Effects of SW on Body and Organ Weights in NAFLD-Induced Mice

**Figure 4:**
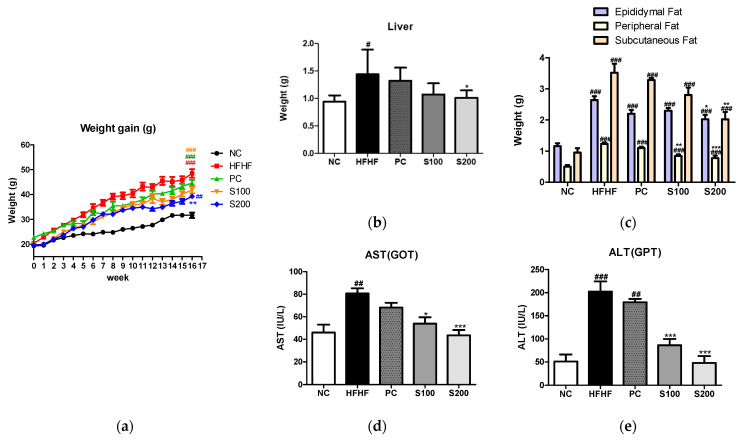
Effects of *Bombyx mori* (SW) on the weight gain, relative tissue weight, and serum AST and ALT in HFHF-fed mice. (**a**) Body weight during the experimental stage. (**b**) Relative liver weight and (**c**) adipose tissues weight. (**d**) Serum AST (GOT) levels. (**e**) Serum ALT (GPT) levels. AST, aspartate transaminase; ALT, alanine transaminase; NC, normal control; HFHF, 45% high fat diet with 10% fructose in the drinking water; PC, positive control (HFHF diet with 1% silymarin); S100, HFHF diet with SW (100 mg/kg/day); S200, HFHF diet with SW (200 mg/kg/day); values are expressed as means ± SE. # *p* < 0.05, ## *p* < 0.01, and ### *p* < 0.001 vs. NC; * *p* < 0.05, ** *p* < 0.01, and *** *p* < 0.001 vs. HFHF.

After the animal experiment, changes in body weight and liver and white adipose tissue weights of the mice were recorded (Figure 4). Upon feeding of an HFHF diet for 16 weeks, the body weights of the HFHF group were significantly increased relative to the control group. However, the S100 (100 mg/kg/day, SW) and S200 (200 mg/kg/day, SW) groups showed reduced weights compared to the HFHF group (Figure 4a). The liver and white adipose tissue weights of mice are presented in Figure 4b,c, respectively. The liver and white adipose tissue weights of the HFHF group were increased relative to the NC group. Meanwhile, the weights of the liver and white adipose tissues of SW-supplemented groups were lower than those of the HFHF group. Serum AST and ALT levels were significantly higher in the HFHF group (Figure 4d,e). Treatment with SW significantly suppressed this increase in the S100 and S200 groups. These results indicate that oral administration of SW reduced the gain of body weight and organ weights and significantly improved liver function.

### 3.6. Effects of SW on Hepatic Steatosis in NAFLD-Induced Mice

**Figure 5:**
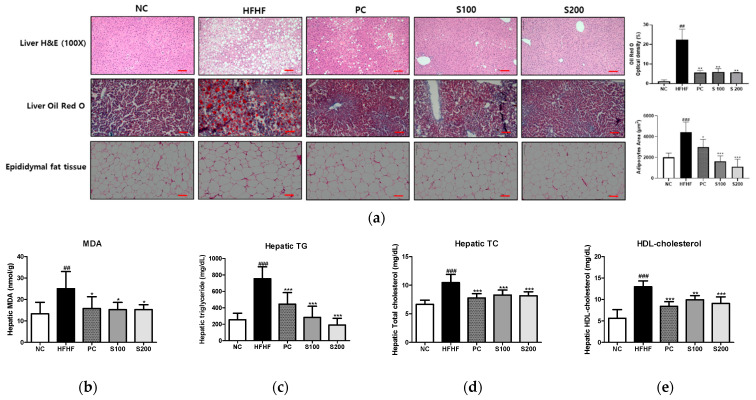
Effects of *Bombyx mori* (SW) on hepatic steatosis and hepatic parameters in HFHF-fed mice. (**a**) Liver tissue histology (100×), Liver Oil Red O, and H&E staining of epididymal white adipose tissue. (**b**) Hepatic MDA levels. The accumulation of liver TG (**c**), TC (**d**), and HDL-cholesterol (**e**). H&E, hematoxylin and eosin; MDA, malondialdehyde; TG, triglyceride; TC, total cholesterol; HDL-cholesterol, high-density lipoprotein-cholesterol; NC, normal control; HFHF, 45% high fat diet with 10% fructose in the drinking water (HFHF) control; PC, positive control (HFHF diet with 1% silymarin); S100, HFHF diet with SW (100 mg/kg/day); S200, HFHF diet with SW (200 mg/kg/day). The results from three independent experiments are expressed as the means ± SE. ## *p* < 0.01 and ### *p* < 0.001 vs. NC; * *p* < 0.05, ** *p* < 0.01, and *** *p* < 0.001 vs. HFHF. The scale bar shows 100 μm.

To investigate the inhibitory effect of SW supplementation on hepatic fat accumulation, we performed histology of the liver tissue using H&E and Oil Red O staining (Figure 5a). In the HFHF group, H&E staining of liver sections showed microvesicular and macrovesicular steatosis within hepatocytes. By contrast, the lipid droplets were significantly reduced in both size and number in the SW-supplemented groups (S100 and S200). Similarly, the HFHF group showed increased liver lipid content, as demonstrated through Oil Red O staining. However, in the S100 and S200 groups, the effect of HFHF on the elevation of lipid content was blocked. Furthermore, the epididymal adipocytes of the HFHF group were larger than those of the NC group but significantly decreased in size after SW treatment.

Hepatic biochemical parameters were measured to identify the preventive effect of SW on hepatic fat accumulation. As shown in Figure 5b–e, the HFHF group showed a significant increase in MDA, hepatic TG, TC, and HDL-cholesterol levels compared to the NC group. The MDA level, which indicates lipid peroxidation, was elevated in the HFHF group relative to the NC group. Compared with the HFHF group, the PC, S100, and S200 groups had significantly reduced MDA, hepatic TG, TC, and HDL- cholesterol levels.

### 3.7. Effects of SW on Hepatic Lipid Gene Expression and Fatty Acid Oxidation in NAFLD-Induced Mice

**Figure 6:**
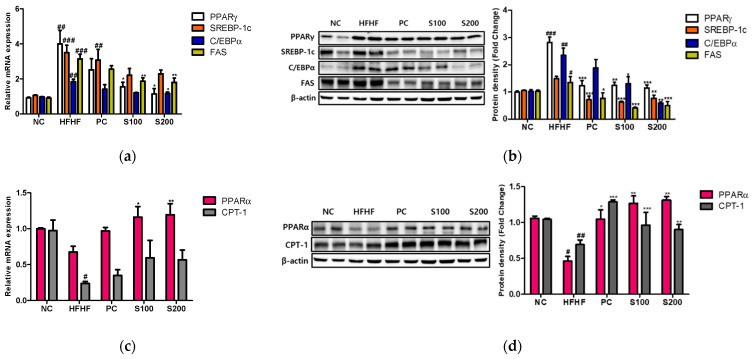
Effects of *Bombyx mori* (SW) on the expression of genes associated with lipogenesis and fatty acid oxidation in HFHF-fed mice. (**a**) Quantitative real-time PCR analysis of PPARγ, SREBP-1c, C/EBPα, and FAS were normalized by β-actin as an internal control. (**b**) Expression of lipogenesis-related proteins, including PPARγ, SREBP-1c, C/EBPα, and FAS. Equal loading of protein was verified by β-actin. (**c**) The mRNA expression of PPARα and CPT-1 were quantified by real-time PCR and normalized by β-actin as an internal control. (**d**) Quantification of the protein levels of PPARα and CPT-1. NC, normal control; HFHF, 45% high fat diet with 10% fructose in the drinking water; PC, positive control (HFHF diet with 1% silymarin); S100, HFHF diet with SW (100 mg/kg/day); S200, HFHF diet with SW (200 mg/kg/day). PPARγ, peroxisome proliferator-activated receptor-gamma; SREBP-1c, sterol regulatory element-binding protein 1-c; C/EBPα, CCAAT/enhancer-binding protein alpha; FAS, fatty acid synthase; PPARα, peroxisome proliferator-activated receptor-alpha; CPT-1, carnitine palmitoyltransferase 1. The results from three independent experiments are expressed as the means ± SE. # *p* < 0.05, ## *p* < 0.01, and ### *p* < 0.001 vs. NC; * *p* < 0.05, ** *p* < 0.01, and *** *p* < 0.001 vs. HFHF.

As shown in Figure 6, the mRNA levels of PPARγ, C/EBPα, SREBP-1c, and FAS and the corresponding protein levels were significantly higher in the HFHF group than in the NC group. However, the expression of PPARγ, C/EBPα, SREBP-1c, and FAS was significantly reduced with SW supplementation (Figure 6a,b). Additionally, the mRNA and immunoblots of PPARα and CPT-1 were decreased in the HFHF group relative to the NC group (Figure 6c,d). Compared to the HFHF group, the protein levels of PPARα and CPT-1 were significantly elevated in response to SW supplementation (Figure 6d).

### 3.8. Effects of SW on the Expression of Phosphorylation of AMPK and ACC in NAFLD-Induced Mice

**Figure 7:**
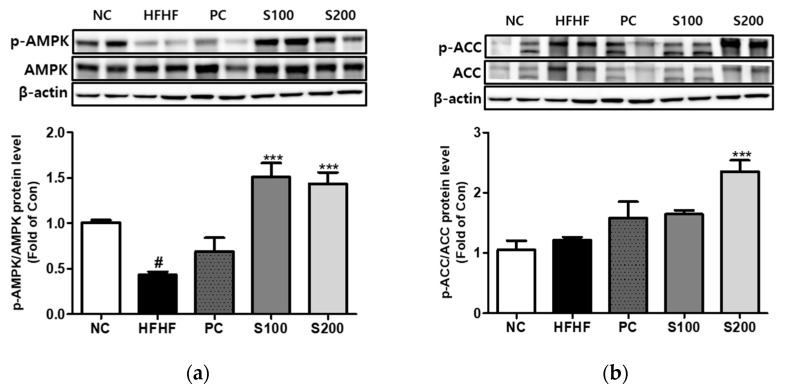
Effects of *Bombyx mori* (SW) on the expression of AMPK and ACC signaling in HFHF-fed mice. The expression of p-AMPK/AMPK (**a**) and p-ACC/ACC (**b**) were quantified by Western blot analysis. AMPK and ACC were used as a protein loading control of phosphorylated AMPK (p-AMPK) and phosphorylated ACC (p-ACC), respectively. β-actin was used as a protein loading control. NC, normal control; HFHF, 45% high fat diet with 10% fructose in the drinking water; PC, positive control (HFHF diet with 1% silymarin); S100, HFHF diet with SW (100 mg/kg/day); S200, HFHF diet with SW (200 mg/kg/day). The results from three independent experiments are expressed as the means ± SE. # *p* < 0.05 vs. NC; *** *p* < 0.001 vs. HFHF.

As shown in Figure 7a,b, the HFHF group showed a reduction in the expression of p-AMPK relative to the NC group. Supplementation with SW restored p-AMPK/AMPK and the p-ACC/ACC ratio to the levels of the HFD group.

### 3.9. Effects of SW on the Composition of Fecal Microbiota

**Figure 8:**
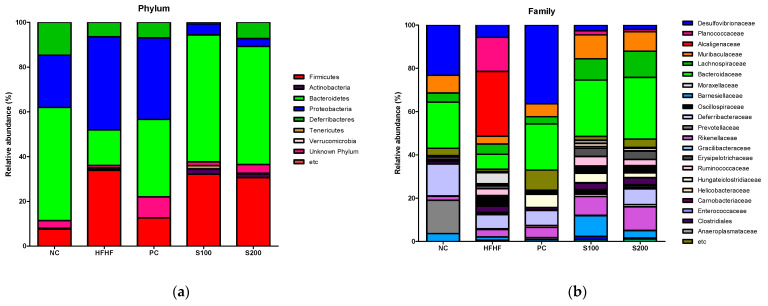
Effects of *Bombyx mori* (SW) treatment on the fecal microbial diversity in HFHF-fed mice. (**a**) Relative abundances of the gut microbiota at phylum level and (**b**) family level. NC, normal control; HFHF, 45% high fat diet with 10% fructose in the drinking water; PC, positive control (HFHF diet with 1% silymarin S100); S100, HFHF diet with SW (100 mg/kg/day); S200, HFHF diet with SW (200 mg/kg/day).

The bacterial composition of fecal samples from the experimental groups was analyzed to investigate the effects of SW on the intestinal microbiota using the Illumina platform. The results are presented as operational taxonomic units (OTUs) and Chao1 richness estimates at the species level (Figure S2A). Shannon and inverse Simpson analyses were used to represent the gut microbial diversity in each sample (Figure S2B). At the OTU and Chao1 levels, the HFHF, S100, and S200 mice had increased numbers of bacterial species compared with NC and PC mice. Diversity, as measured with Shannon’s richness index and inverse Simpson’s evenness index, was reduced in the PC group compared with the NC group. SW treatment increased Shannon’s index and inverse Simpson’s evenness index values relative to the other groups (NC, PC, and HFHF). As shown in Figure 8a,b, significant differences in gut microbial composition were found through taxon-based analysis. At the phylum level, Bacteroidetes, Proteobacteria, Deferribacteres, and Firmicutes were the most prevalent bacterial communities in the NC group. The HFHF group showed dramatically differing abundances of Bacteroidetes (50.59% vs. 15.89%), Proteobacteria (23.36% vs. 41.62%), Deferribacteres (14.67% vs. 6.44%), and Firmicutes (7.50% vs. 33.84%) compared with the NC group. At the family level, the PC and SW groups showed higher abundances of Bacteroidaceae (bright green color in Figure 8b, phylum Bacteroidetes) and lower numbers of Planococcaceae (phylum Firmicutes) and Alcaligenaceae (phylum Proteobacteria) relative to the HFHF group. Additionally, the relative abundance of Lachnospiraceae (dark green color in Figure 8b, phylum Firmicutes) was more abundant in the S100 and S200 groups than the HFHF group.

## 4. Discussion

NAFLD is one of the leading causes of chronic liver disease worldwide and will probably emerge as a major cause of end-stage liver disease within the next few decades [35,36]. Non-alcoholic fatty steatohepatitis (NASH), an aggressive form of NAFLD, is the leading cause of liver cirrhosis and hepatocellular carcinoma [32]. Moreover, individuals with NAFLD have a high frequency of metabolic complications, representing an increasing burden on healthcare systems [33].

As the population grows, edible insects could be a new food source to supplement insufficient food supplies and reduce undernourishment and toxicology [34,37]. Insect cells are also a promising source of high-quality protein that is highly digestible and contains high essential amino acid concentrations [34]. The silkworm, which can be used medicinally as a health supplement, has adequate proportions of amino acids and unsaturated fatty acids to meet human nutritional requirements [38]. Hong et al. [39] reported the protective effects of steamed and freeze-dried mature silkworm larval powder (SMSP) on ethanol-induced hepatic steatosis, oxidative stress, inflammation, and lipid metabolism in rats. Additionally, he suggested that SMSP supplementation could be a new approach for the prevention and/or treatment of chronic alcoholic fatty liver disease. SW consists mainly of 70% protein of fibroin and sericin and is a recurrent structure of amino acid serine, glycine, alanine, tyrosine, and threonine [40]. The rise in serine levels in hepatocytes has been reported to be beneficial for patients with non-alcoholic fatty liver disease by synthesizing sphingosine [41].

In this study, we investigated the therapeutic mechanism of SW for the treatment of NAFLD, using the in vitro cellular model of hepatic steatosis in HepG2 cells and the in vivo model of HFHF-fed mice. We examined changes in cellular lipid droplets using Oil Red O staining and expression of genes involved in lipid metabolism. In this study, SW treatment significantly reduced lipid accumulation and the expression levels of PPARγ, C/EBPα, SREBP-1c, and FAS in both in vitro and in vivo in a concentration-dependent manner. The HFHF group showed widespread fat droplet vacuoles and red lipid droplets in hepatocytes, while no such deposits were observed in the NC group. The accumulation of lipids in the liver results from an imbalance between lipid deposition and the elimination of lipids based on dietary intake, the hepatic synthesis of triglycerides, and de novo lipogenesis [42]. Excessive accumulation of lipids in liver cells involves various organizational changes ranging from “simple” steatosis to non-alcoholic steatohepatitis as time progresses [5]. The HFHF-fed mice showed significant increases in body weight, as well as liver and white adipocyte tissue weights, after 16 weeks of induced obesity. However, SW treatment for 8 weeks significantly reduced the body, liver, and white adipocyte tissue weights; serum AST, ALT, and hepatic MDA levels; and hepatic index values affected by HFHF. Weight loss associated with dietary changes plays a vital role in NAFLD treatment [43]. Consistent with the in vitro hepatocyte experiments, SW supplementation significantly reduced lipid droplets and decreased epididymal fat tissue size in the HFHF-fed mice. These results suggest that a nutritional supplement made from SW could be a new therapeutic option for treating hepatic steatosis related to obesity.

A characteristic of NAFLD is the accumulation of triglycerides in the cytoplasm of hepatocytes, which is caused by an imbalance between lipid acquisition and removal [4]. An increasing expression level of hepatic CD36 has been observed in NAFLD, and appears to mediate enhanced intake of free fatty acids derived from the plasma through de novo lipogenesis [44,45]. The balance of hepatic triglycerides is also affected by mitochondrial β-oxidation, which is essential for the production of ATP and ketone bodies [46]. Rearrangement of fatty acyl-CoAs in the outer mitochondrial membrane is linked with the conversion to acyl-carnitines by CPT-1 [4]. Therefore, overexpression of CPT-1 is sufficient to enhance fatty acid oxidation in mice fed a high-fat diet [47]. In our study, SW treatment led to upregulated expression of CPT-1 and PPARα in HepG2 cells and HFHF-fed mice. PPARα promotes liver regeneration by regulating the cell cycle, while increasing fatty acid oxidation and lipase activity. It also reduces systemic lipid load when liver lipid metabolism is damaged, resulting in protection against fasting-induced hepatosteatosis [48,49].

SW treatment induced increases in AMPK and ACC phosphorylation, of which the latter is a central enzyme involved in fatty acid β-oxidation and the former is a master regulator of metabolism in multiple tissues that promotes fatty acid oxidation through phosphorylation of ACC while also regulating fatty acid synthesis [50,51]. As the role of the AMPK-ACC pathway is to regulate fatty acid synthesis, AMPK activation is an essential process in conditions related to increased fatty acid production, such as NAFLD [29,52]. In addition, AMPK inhibits the FAS protein level and activation of SREBP-1c, which is a major transcriptional regulator of fatty acid synthesis. Activation of SREBP-1c leads to severe metabolic conditions such as obesity, type 2 diabetes, and hepatosteatosis, and inflammation and fibrosis in various organs [53]. The phosphorylation and gene expression of SREBP-1c were inhibited by AMPK in hepatocytes exposed to high glucose, thereby reducing lipid accumulation [54]. We suggest that SW shows antiobesity effects in HFHF-fed mice.

The interaction between dietary components and gut microbiota leads to changes in the intestinal microbial composition, which dramatically affect host metabolism. The fecal microbiota of the SW group showed an increased proportion of Bacteroidetes and a decreased proportion of Firmicutes, similar to results for silymarin-treated positive control mice. Mouzaki et al. [55] found a significant association between liver health and lower percentages of Bacteroidetes. Obese diabetic *db/db* mice showed substantial decreases in the abundance of Bacteroidetes and decreased abundances of Firmicutes [56]. Additionally, the administration of Lachnospiraceae, Roseburia spp. to alcohol-related liver diseases (ALDs) has significantly improve both hepatic steatosis and inflammation [57]. In this study, there is a limit to describing detailed species about the improvement of hepatic steatosis in NAFLD-induced model mice, and we need additional study to describe the relationship between gut microbiome and NAFLD.

This study provides evidence that SW reduces lipid accumulation and inhibits the expression of PPARγ, C/EBPα, SREBP-1c, and FAS by increasing the phosphorylation of AMPK and ACC based on in vitro and in vivo experiments. Daily supplementation with SW attenuated the body weight gain, lipid droplet accumulation, serum AST and ALT levels, and hepatic TG level of HFHF-diet mice. We suggest that SW might be a new therapeutic agent for preventing or reversing hepatic steatosis by targeting hepatic AMPK and ACC signaling mediated lipid metabolism.

## Data Availability

Not applicable.

## Supplementary Materials

The following are available online at https://www.mdpi.com/article/10.3390/cimb43010003/s1. Figure S1: Metabolomic profiling of steam-boiled SW powder samples using GC-TOF-MS (A) and UHPLC-LTQ-Orbitrap-MS/MS (B). Figure S2: Responses of the diversity and richness of the gut microbiota to Bombyx mori (SW) during the treatment of obesity in mice. Table S1: Metabolites identified in a steam-boiled silkworm powder sample by GC-TOF-MS. Table S2: Metabolites identified in a steam-boiled silkworm powder sample by UHPLC-LTQ-Orbitrap-MS. Table S3: The primer sets used for real-time PCR in this study.
