# Supplementary material for: Effect of Bombyx mori on the Liver Protection of Non-Alcoholic Fatty Liver Disease Based on In Vitro and In Vivo Models
Source: Curr Issues Mol Biol. 2021 Apr 28;43(1):21–35. doi: 10.3390/cimb43010003 (PMC8929127; doi:10.3390/cimb43010003)
Supplement: Supplementary file 1 [file cimb-43-00003-s001.zip › Supplement Tables.pdf]

Table S1. Metabolites identified in a steam-boiled silkworm powder sample by GC-TOF-MS.

No.	Tentative identifications[a]	tR[b] (min:sec)	Unique mass (*m/z*)	MS[c] fragment patternc (m/z)	TMS[d]	ID[e]
Amino acids						
1	Valine	06:55.8	144	144, 73, 218, 147, 145, 100, 59, 74, 75, 146	2	STD MS
2	Serine	08:19.3	204	73, 204, 218, 75, 205, 116, 74, 219, 206, 278	3	STD
3	Threonine	08:34.2	117	73, 218, 117, 219, 101, 147, 57, 75, 74, 291	3	STD
4	β-Alanine	08:54.6	174	174, 73, 248, 147, 86, 290, 100, 59, 133, 175, 249	3	MS
5	Aspartic acid	09:19.9	232	73, 232, 70, 147, 100, 75, 233, 133, 74, 59, 148	3	STD MS
6	Pyroglutamic acid	09:46.2	156	156, 73, 147, 157, 74, 230, 258, 72, 58, 75, 231	2	MS
7	Glutamic acid	10:28.7	246	73, 246, 128, 147, 75, 247, 156, 84, 230, 56	3	MS
8	Asparagine	10:54.0	116	73, 116, 231, 132, 147, 74, 75, 141, 100, 188	3	STD MS
9	Lysine	12:39.7	156	73, 174, 156, 317, 128, 318, 230, 100, 86, 175	4	STD MS
10	Tyrosine	12:48.1	218	218, 73, 219, 100, 280, 220, 179, 74, 281	3	STD MS
Sugar and Sugar derivatives						
11	Glyceric acid	08:02.8	189	73, 147, 189, 103, 133, 292, 102, 117, 74, 205	3	MS
12	Carbohydrate 1	10:59.7	103	73, 103, 147, 217, 205, 307, 117, 74, 133, 173	–	MS
13	Carbohydrate 2	11:29.4	205	73, 147, 205, 217, 117, 74, 148, 133, 394, 206	–	MS
14	Ribonic acid	11:43.7	103	73, 147, 103, 292, 217, 74, 205, 128, 189, 133	5	MS
15	D-Glucose	12:36.2	205	73, 205, 73, 319, 147, 160, 103, 217, 117, 320	5	STD MS
16	Carbohydrate 3	12:51.8	319	73, 147, 319, 205, 217, 74, 103, 117, 129, 204, 148	–	MS
17	myo-Inositol	13:51.5	217	73, 217, 147, 305, 191, 318, 306, 204, 218, 129, 319	6	STD MS
18	Carbohydrate 4	14:05.3	319	73, 319, 147, 205, 103, 74, 320, 133, 217, 117	–	MS
19	Glyceryl-glycoside	15:06.8	204	204, 73, 147, 217, 103, 205, 129, 206, 337	6	MS
Fatty acids						
20	Stearic acid	14:32.9	117	117, 73, 75, 132, 129, 341, 145, 55, 342	1	STD MS
21	Oleamide	15:32.8	131	75, 131, 144, 73, 116, 128, 55, 338, 69, 353, 115, 145	1	MS
Etc.						
22	Butanediol	04:55.6	117	117, 73, 147, 75, 118, 74, 133, 66, 148, 119	2	MS

23	Hydroxylamine	05:52.1	133	73. 133. 146. 119. 59. 147. 249. 130	3	MS
24	Pyruvic acid	06:08.9	133	73, 147, 133, 59, 100, 72, 148, 220, 74, 86, 235	2	MS
25	Urea	07:06.8	189	147, 73, 189, 171, 99, 148, 75, 14, 131, 59	2	MS
26	Hydroxybenzoic acid	10:32.7	267	267, 223, 193, 282, 268, 126, 269, 224, 194, 283	2	STD MS
27	α-Glycerophosphoric acid	11:35.5	299	73, 299, 357, 147, 101, 103, 133, 211, 129	4	MS
28	1-Deoxynojirimycin	12:09.0	420	420, 216, 147, 421, 217, 422, 129, 133, 218	–	STD
29	Pantothenic acid	13:09.6	291	75, 291, 157, 117, 201, 247, 159, 129, 55, 144, 420	3	MS
30	Phytol	14:09.9	143	143, 73, 75, 144, 123, 57, 55, 81, 69, 103	1	MS
Non-Identifications						
31	N.I. 1	04:06.4	171	171, 73, 172, 78, 173, 64, 151, 186, 100	–	–
32	N.I. 2	04:34.0	89	73, 89, 59, 161, 74, 90, 60, 58, 75, 91	–	–
33	N.I. 3	05:02.0	123	123, 93, 55, 125, 95, 103, 59, 124	–	–
34	N.I. 4	06:17.2	86	86, 75, 73, 87, 74, 188, 146, 103, 70, 61, 170, 76	–	–
35	N.I. 5	06:31.0	86	86, 75, 73, 69, 87, 74, 146, 57, 56, 188, 103	–	–
36	N.I. 6	08:15.4	57	57, 73, 97, 127, 215, 111, 54, 109, 69,, 216, 246, 159	–	–
37	N.I. 7	09:33.0	84	84, 75, 158, 157, 186, 73, 85, 56, 116, 103	–	–
38	N.I. 8	09:40.2	131	73, 131, 147, 75, 306, 74, 133, 130, 132, 259	–	–
39	N.I. 9	09:55.7	205	205, 292, 147, 220, 217, 102, 232, 293, 221, 142, 206, 149	–	–
40	N.I. 10	10:03.4	117	73, 147, 292, 117, 205, 217, 103, 74, 220, 133	–	–
41	N.I. 11	10:07.3	129	73, 129, 147, 247, 75, 203, 157, 85, 149, 133, 349	–	–
42	N.I. 12	10:15.7	245	73, 147, 245, 75, 74, 83, 148, 133, 149	–	–
43	N.I. 15	10:56.9	129	73, 147, 75, 129, 74, 149, 133, 109, 247, 148, 363	–	–
44	N.I. 16	11:02.9	260	73, 144, 260, 147, 170, 129, 116, 75, 128, 145, 261	–	–

[a] Tentative metabolites based on variable important projection (VIP) analysis with a cutoff value of 0.7 and *p*-value < 0.05. [b] Retention time; [c] MS fragment patterns detected
[d] Trimethylsilyl; [e] Identification: STD, Standard

Table S2. Metabolites identified in a steam-boiled silkworm powder sample by UHPLC-LTQ-Orbitrap-MS.

No.	tR[a] (min)	Metabolites[b]	$[M–H]^–$	M.W.[c]	MS[d] Fragments pattern (m/z)	Molecular Formula	Δ ppm	REF[e]
Carboxylic acids								
1	0.92	Malic acid	133.0150	134	115,87>71	C4H6O5	5.289	(1)
2	1.05	Citric acid	191.0202	192	111>66	C6H8O7	2.639	(2), LIB
Hydroxybenzoic acids								
3	1.36	Pantothenic acid	218.1041	219	146>88>59	C9H17NO5	3.412	(3), LIB
4	1.44	Gentisoyl hexoside	315.0726	316	153>109	C13H16O9	1.507	(2, 4)
Phenolic acids								
5	0.84	Quinic acid	191.0568	192	173,127,111,85,170>113,143	C7H12O6	3.343	(1)
6	3.65	*p*-Coumaric acid	163.0408	164	119	C9H8O3	4.248	(2, 5)
7	3.87	Caffeoylquinic acid	353.0883	354	191,173>126,85	C16H18O9	1.288	(5), LIB
8	4.44	Coumaroylquinic acid	337.0936	338	191,163>172,126,85	C16H18O8	2.193	(2), LIB
Flavonols								
9	4.01	Quercetin-hexosyl hexoside	625.1419	626	463,301>300,445>178,150,273,270	C27H30O17	1.420	(2, 5), LIB
10	4.03	Quercetin-rhamnosyl dihexoside	771.1993	772	609,300>301>270,178,150,255	C33H40O21	0.530	(6)
11	4.23	Kaempferol-rutinoside-hexoside	755.2051	756	593>285>257	C33H40O20	1.488	(1, 2)
12	4.58	Quercetin-rhamnose-hexose-rhamnose	755.2044	756	300>271,255>243,227	C33H40O20	0.442	(1)
13	4.72	Kaempferol O-rhamnosyl rutinoside	739.2111	740	575,284,255>339,393,429,309,547>311	C33H40O19	2.730	(7)
14	4.84	Rutin	609.1480	610	301>271,255>243,227	C27H30O16	3.123	(1, 5), LIB
15	4.96	Isoquercitrin	463.0892	464	301>178,150,271,255>150	C21H20O12	2.161	(5)
16	5.03	Kaempferol-rhamnosyl hexoside	593.1517	594	285,284>255,277,211	C27H30O15	0.905	(2, 5)
17	5.10	Quercetin-malonyl hexoside	549.0892	550	505>301,463>271,178,150,255	C24H22O15	1.160	(1)
18	5.19	Kaempferol-hexoside	447.0945	448	285>255>227	C21H20O11	2.674	(5), LIB

Lysophospholipids								
19	8.22	LysoPC (18:3)	562.3160	517	502, 277>233,259	C26H48O7NP	1.669	(8)
20	8.46	LysoPE(18:2)	476.2795	477	279>261>243, 233	C23H44NO7P	2.619	(8)
21	8.65	LysoPC (18:2)	564.3313	519	504>279>261	C26H50NO7P	1.078	(8, 9)
22	8.76	LysoPE (16:0)	452.2792	453	255>237	C21H44NO7P	2.007	(8)
23	9.00	LysoPC (16:0)	540.3313	495	480>255>237	C24H50NO7P	1.126	(8)
24	10.07	LysoPC (18:0)	568.3629	523	508>283>265	C26H54NO7P	2.372	(7)
Etc.								
25	0.82	Maltose	341.1105	342	179,161>160,142,88	C12H22O11	4.501	(3)
26	1.95	Protocatechuic acid	153.0203	154	108>81	C7H6O4	-0.013	(5, 6)
27	6.63	9,12,13-TriHOME	329.2343	330	229, 311,171>211,292>183, 274	C18H34O5	2.742	(7)

[a] Retention time. [b] Tentative metabolites based on variable important projection (VIP) analysis with a cutoff value of 0.7 and p-value < 0.05. [c] Molecular weight [d] MS^n fragment patterns detected in the negative ion mode. [e] Reference LIB, in house Library [f] Lysophosphatidylcholine [g] lysophosphatidylethanolamine

Table S3. The primer sets used for real-time PCR in this study.

	Gene	Forward (5' – 3')	Reverse (5' – 3')
HepG2	PPARγ	TGCAGGTGATCAAGAAGACG	AGTGCAACTGGAAGAAGGGA
	C/EBPα	TGGACAAGAACAGCAACGAGTA	ATTGTCACTGGTCAGCTCCAG
	SREBP-1c	GCGCCTTGACAGGTGAAGTC	GCCAGGGAAGTCACTGTCTTG
	FAS	CCCCTGATGAAGAAGGATCA	ACTCCACAGGTGGGAACAAG
	PPARα	ACGATTCGACTCAAGCTGGT	GTTGTGTGACATCCCGACAG
	CPT-1	CCTCCGTAGCTGACTCGGTA	GGAGTGACCGTGAACTGAAA
	β-actin	CTCTTCCAGCCTTCCTTCCT	AGCACTGTGTTGGCGTACAG
Mouse Liver	PPARγ	CAGGAGAGCAGGGATTTGCA	CCTACGCTCAGCCCTCTTCAT
	C/EBPα	TTACAACAGGCCAGGTTTCC	GGCTGGCGACATACAGTACA
	SREBP-1c	ATCGCAAACAAGCTGACCTG	AGATCCAGGTTTGAGGTGGG
	FAS	TTGCTGGCACTACAGAATGC	AACAGCCTCAGAGCGACAAT
	PPARα	CAGGAGAGCAGGGATTTGCA	CCTACGCTCAGCCCTCTTCAT
	CPT-1	CTCAGTGGGAGCGACTCTTCA	GGCCTCTGTGGTACACGACAA
	β-actin	CTGTCCCTGTATGCCTCTG	ATGTCACGCACGATTTCC

PPARγ, peroxisome proliferator-activated receptor gamma; C/EBPα, CCAAT/enhancer-binding protein alpha; SREBP-1c, sterol regulatory element-binding protein1-c; FAS, fatty acid synthase; CPT-1, carnitine palmitoyltransferase-1; PPARα, Peroxisome proliferator-activated receptor alpha

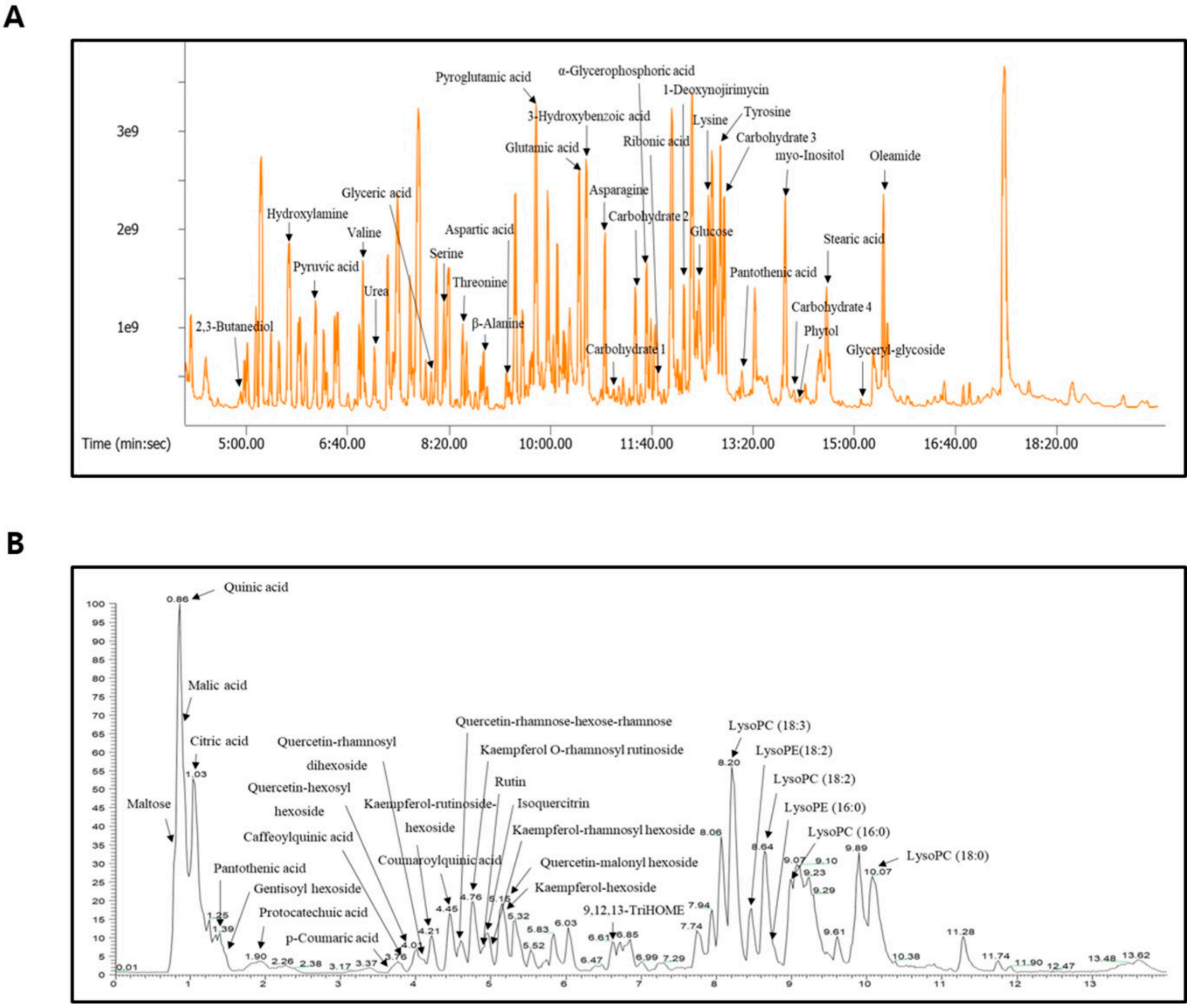

Figure S1. Metabolomic profiling of steam-boiled SW powder samples using GC-TOF-MS (A) and UHPLC-LTQ-Orbitrap-MS/MS (B).

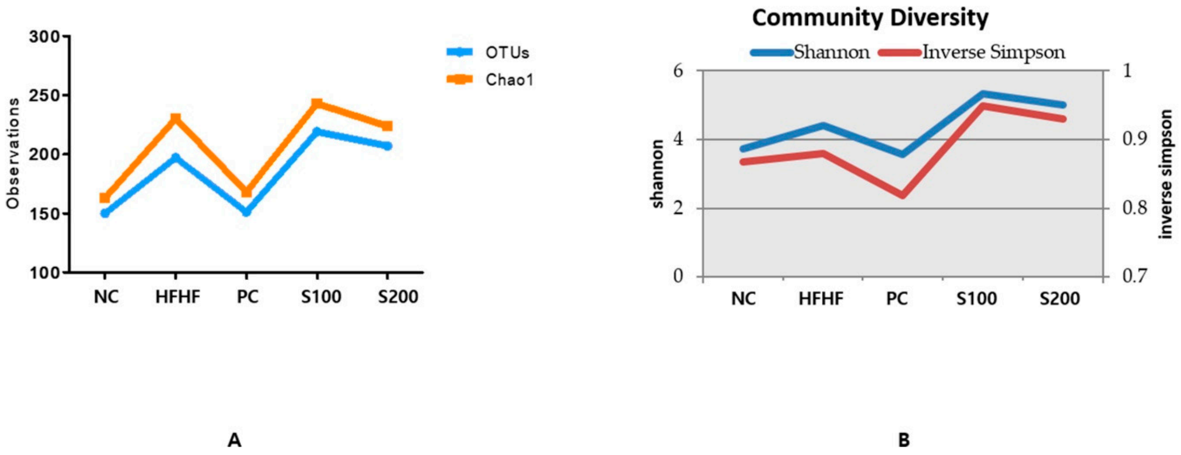

Figure S2. Responses of the diversity and richness of the gut microbiota to *Bombyx mori* (SW) during the treatment of obesity in mice. (a) Operational taxonomic units (OUTs) and Chao1 number of gut microbiota in five groups. (b) Shannon curves and inverse simpson curves of gut microbiota for each group, respectively.

References

1. Sánchez-Salcedo EM, Tassotti M, Del Rio D, Hernández F, Martínez JJ, Mena P. (Poly)phenolic fingerprint and chemometric analysis of white (Morus alba L.) and black (Morus nigra L.) mulberry leaves by using a non-targeted UHPLC–MS approach. Food Chemistry. 2016 2016/12/01/;212:250-5.
2. Mena P, Sánchez-Salcedo EM, Tassotti M, Martínez J, Hernández F, Rio D. Phytochemical evaluation of eight white (Morus alba L.) and black (Morus nigra L.) mulberry clones grown in Spain based on UHPLC-ESI-MSn metabolomic profiles. Food Research International. 2016;89:1116-22.
3. Yang J, Wen H, Zhang L, Zhang X, Fu Z, Li J. The influence of ripening stage and region on the chemical compounds in mulberry fruits (Morus atropurpurea Roxb.) based on UPLC-QTOF-MS. Food Research International. 2017 2017/10/01/;100:159-65.
4. Cao X, Yang L, Xue Q, Yao F, Sun J, Yang F, Liu Y. Antioxidant evaluation-guided chemical profiling and structure-activity analysis of leaf extracts from five trees in Broussonetia and Morus (Moraceae). Scientific Reports. 2020 2020/03/16;10:4808.
5. Natić MM, Dabić D, Papetti A, Fotirić Akšić MM, Ognjanov V, Ljubojević M, Tešić Ž. Analysis and characterisation of phytochemicals in mulberry (Morus alba L.) fruits grown in Vojvodina, North Serbia. Food Chem. 2015 Mar 15;171:128-36.
6. Jin Q, Yang J, Ma L, Wen D, Chen F, Li J. Identification of polyphenols in mulberry (genus Morus) cultivars by liquid chromatography with time-of-flight mass spectrometer. Journal of Food Composition and Analysis. 2017 2017/10/01/;63:55-64.
7. Son SY, Lee S, Singh D, Lee NR, Lee DY, Lee CH. Comprehensive Secondary Metabolite Profiling Toward Delineating the Solid and Submerged-State Fermentation of Aspergillus oryzae KCCM 12698. Frontiers in microbiology. 2018;9:1076.
8. Lee DE, Lee S, Singh D, Jang ES, Shin HW, Moon BS, Lee CH. Time-resolved comparative metabolomes for Koji fermentation with brown-, white-, and giant embryo-rice. Food Chem. 2017 Sep 15;231:258-66.
9. Shin GR, Lee S, Lee S, Do S-G, Shin E, Lee CH. Maturity stage-specific metabolite profiling of Cudrania tricuspidata and its correlation with antioxidant activity. Industrial Crops and Products. 2015 2015/08/01/;70:322-31.
